# SLAMF8 Participates in Acute Renal Transplant Rejection *via* TLR4 Pathway on Pro-Inflammatory Macrophages

**DOI:** 10.3389/fimmu.2022.846695

**Published:** 2022-04-01

**Authors:** Lisha Teng, Lingling Shen, Wenjun Zhao, Cuili Wang, Shi Feng, Yucheng Wang, Yan Bi, Song Rong, Nelli Shushakova, Hermann Haller, Jianghua Chen, Hong Jiang

**Affiliations:** ^1^Kidney Disease Center, The First Affiliated Hospital, College of Medicine, Zhejiang University, Hangzhou, China; ^2^Key Laboratory of Nephropathy, Hangzhou, China; ^3^Institute of Nephropathy, Zhejiang University, Hangzhou, China; ^4^Zhejiang Clinical Research Center of Kidney and Urinary System Disease, Hangzhou, China; ^5^Department of Nephrology, Hannover Medical School, Hannover, Germany

**Keywords:** acute rejection, renal transplantation, weighted gene co-expression network analysis (WGCNA), hub gene, SLAMF8, gene set enrichment analysis

## Abstract

**Background:**

Acute rejection (AR) in kidney transplantation is an established risk factor that reduces the survival rate of allografts. Despite standard immunosuppression, molecules with regulatory control in the immune pathway of AR can be used as important targets for therapeutic operations to prevent rejection.

**Methods:**

We downloaded the microarray data of 15 AR patients and 37 non-acute rejection (NAR) patients from Gene Expression Omnibus (GEO). Gene network was constructed, and genes were classified into different modules using weighted gene co-expression network analysis (WGCNA). Kyoto Encyclopedia of Genes and Genomes (KEGG) and Cytoscape were applied for the hub genes in the most related module to AR. Different cell types were explored by xCell online database and single-cell RNA sequencing. We also validated the SLAMF8 and TLR4 levels in Raw264.7 and human kidney tissues of TCMR.

**Results:**

A total of 1,561 differentially expressed genes were filtered. WGCNA was constructed, and genes were classified into 12 modules. Among them, the green module was most closely associated with AR. These genes were significantly enriched in 20 pathway terms, such as cytokine–cytokine receptor interaction, chemokine signaling pathway, and other important regulatory processes. Intersection with GS > 0.4, MM > 0.9, the top 10 MCC values and DEGs in the green module, and six hub genes (DOCK2, NCKAP1L, IL2RG, SLAMF8, CD180, and PTPRE) were identified. Their expression levels were all confirmed to be significantly elevated in AR patients in GEO, Nephroseq, and quantitative real-time PCR (qRT-PCR). Single-cell RNA sequencing showed that AR patient had a higher percentage of native T, CD1C+_B DC, NKT, NK, and monocytes in peripheral blood mononuclear cells (PBMCs). Xcell enrichment scores of 20 cell types were significantly different (p<0.01), mostly immune cells, such as B cells, CD4+ Tem, CD8+ T cells, CD8+ Tcm, macrophages, M1, and monocytes. GSEA suggests that highly expressed six hub genes are correlated with allograft rejection, interferon γ response, interferon α response, and inflammatory response. In addition, SLAMF8 is highly expressed in human kidney tissues of TCMR and in M1 phenotype macrophages of Raw264.7 cell line WGCNA accompanied by high expression of TLR4.

**Conclusion:**

This study demonstrates six hub genes and functionally enriched pathways related to AR. SLAMF8 is involved in the M1 macrophages *via* TLR4, which contributed to AR process.

## Introduction

Kidney transplantation is the most optimal renal replacement therapy both for the quality and quantity of life that it provides and for cost effectiveness compared to classic maintenance dialysis for patients in end-stage renal disease (ESRD) (1, 2). The overall risk of acute rejection within 1 year after transplantation has been steadily decreasing to <15% with the introduction of several newer immunosuppressive agents. Nevertheless, short-term improvement in graft survival decreased since 2000, and disappointingly, long-term improvement remained unchanged (3–6). Timing of acute rejection (AR) and the number of episodes still remains a major risk factor for the development of chronic renal allograft failure (CAF), which is a major cause of late graft loss (7–9). AR consists of two distinct diseases: T-cell-mediated rejection (TCMR) characterized by arteritis, interstitial inflammation, and tubulitis, which is the main type in the first year of AR and antibody-mediated rejection (ABMR) refined to encompass histological evidence of capillaritis and serological evidence (10, 11). However, as far as we know, the pathophysiology of AR is multifactorial and still not fully defined. Therefore, there is a continuing need to screen new biomarkers for the diagnosis and treatment of allograft rejection after kidney transplantation.

Transcriptional genomic information to acute allograft rejection after renal transplantation has shed new light on our understanding of the pathogenesis (12). Weighted gene co-expression network analysis (WGCNA) has been applied to many important studies such as cancer (13, 14), autoimmune diseases (15, 16), and neurodegenerative diseases (17, 18) since its introduction in 2005. WGCNA can potentially identify the gene network significantly involved in AR, and hub gene in estimating network structures can improve the performance of the predicting biological processes and gene regulation to get deep understanding of its pathogenesis (19). Two recent studies identified several genes associated with kidney transplant rejection *via* WGCNA based on peripheral blood lymphocytes (PBLs) or peripheral blood (PB) (20, 21). Nevertheless, there are few relative studies on kidney transplantation based on percutaneous allograft biopsy.

Due to the latest advances in basic science, macrophages serve as crucial mediators of acute and chronic allograft immunopathology. It is well known that macrophages can trigger an adaptive immune response, persist T-cell-mediated rejection and antibody-mediated rejection, and promote allograft fibrosis (22). Renal macrophages exhibit a pro-inflammatory phenotype signature for interferon gamma (IFNγ) activated and secrete a variety of cytokines, which can activate endothelial cells and promote the production of cytotoxic T cells during acute TCMR associated with poor allograft outcomes (23, 24). Therefore, macrophages have important effects on transplantation results. However, the exact mechanisms controlling macrophage functions are not yet completely understood.

In this study, by using WGCNA-based methods, we downloaded the Gene Expression Omnibus database GSE138043 and screened six hub genes related to the AR. Kyoto Encyclopedia of Genes and Genomes (KEGG) enrichment analysis was performed to reveal pathways in target module, which possibly influence the pathogenesis of AR, and Gene Set Enrichment Analysis (GSEA) was performed to show enrichment results of differentially expressed genes in six hub genes high-expression groups. In addition, we utilized PBMC from patient in whom acute rejection occurred after surgery in our hospital to validate six hub genes. We performed single-cell RNA sequencing to further study the cell types changes related to AR. The AR patient had a higher percentage of native T, CD1C+_B DC, NKT, NK, and monocytes. Immunohistochemistry of SLAMF8 revealed that SLAMF8^+^ cells infiltrated in the human allograft tissue in AR. We constructed Immunofluorescence staining of SLAMF8 and TLR4 to validate that SLAMF8 was involved in the pro-inflammatory macrophages *via* TLR4, which contributed to AR process *in vivo* and *in vitro.*

## Materials and Methods

### Data Collection and Preprocessing

We downloaded mRNA expression profiles of human AR from the Gene Expression Omnibus (GEO) database. In our study, GSE138043 was used to construct co-expression networks and identify hub genes related to AR. The microarray dataset provided gene expression profile in the percutaneous allograft biopsy from 15 AR patients and 37 NAR (25). According to the data processing information of GSE138043, each dataset was normalized independently using Robust Multiarray Average (RMA) followed by log2 transformation and quantile normalization. Data from GSE50058 and GSE343 were used for hub genes validation. In the GSE50058 dataset, 42 AR patients and 58 STA individuals were recruited, and the RNA was extracted from their renal allograft biopsy. In the GSE343 dataset, the total RNA was extracted from the kidney tissue of 25AR patients and 15 NAR. **Supplementary Table S5** shows the summary of discovery and validation microarray data sets of clinical biopsy samples from kidney transplants.

### Differentially Expressed Genes Screening

“limma” R package was utilized to the differentially expressed screen genes (DEGs) between AR and NAR in the expressing data. The genes with adjusted p-value <0.05 were selected as having significant change. “ggplot2” and “pheatmap” were used respectively to paint the volcano plot and heatmap of all DEGs.

### Construction of Co-expression Network

The co-expression network of the genes was constructed based on GSE138043 microarray dataset by the R package “WGCNA.” The soft-thresholding power that we chose was 17 when 0.9 was used as the correlation coefficient threshold. We defined 0.25 as the threshold for cut height to merge possible similar modules.

### Functional Enrichment Analysis

To obtain further insights into the function of the target module most related to AR, we referred to the Database for Annotation, Visualization and Integrated Discovery (DAVID) (https://david.ncifcrf.gov/) to perform the KEGG enrichment analysis. The results were shown graphically by the R package “ggplot2.”

### Hub Genes Identification

The green module, which was most significantly related to AR, was imported into Cytoscape with their weighted correlations. We identified the hub gene with the following criteria: (1) DEGs in green module; (2) gene significance (GS) > 0.4 and module membership (MM) > 0.9; (3) and top 10 Maximal Clique Centrality (MCC)value calculated by the Cytohubba package in Cytoscape v3.8.2.

### Single-Cell RNA Sequencing

A 10X Genomics Chromium machine was used for single-cell capture and cDNA preparation following manufacture’s instruction. Chromium™ Single Cell 3′ Solution was used to perform reverse transcription on gel bead in emulsion, followed by cDNA cleanup and amplification. The cDNA is digested and broken into fragments of about 200–300 bp, followed by the traditional second-generation sequencing library construction process, and PCR amplification is performed to obtain a DNA library. Illumina sequencing platform of paired-end sequencing mode was used to perform high-throughput sequencing on the established library. Sequence data were processed with Cell Ranger V2.1.0 (10X Genomics).

### Quality Control

Then, quality control was performed to filter low-quality cells. For 10X-derived datasets, we only retained cells that had (1) genes more than 200 and <6,000, (2) UMIs more than 500 and <40,000, and (3) <15% of reads mapped to mitochondrial genes.

### Clinical Validation

The Nephroseq v5 online database (http://v5.nephroseq.org/), an integrated data-mining platform for gene expression data sets of kidney diseases, was adopted to validate the correlation between the hub genes and clinical manifestations of AR by Spearman rank correlation coefficient analysis. A p-value of <0.05 was considered statistically significant. r>0.6 was considered strong correlation, and 0.4<r<0.6 was considered medium intensity correlation.

### Cell Types Analysis

The xCell (https://xcell.ucsf.edu/), which is a webtool that performs cell-type enrichment analysis from gene expression data for 64 immune and stroma cell types, was adopted to reveal different infiltrating cell types between AR and NAR, and adjusted p-value < 0.01 was chosen as the cutoff criterion. Cell-type enrichment score is shown in **Supplementary Table S3**. To further explore the six hub genes expression in 76 single cell types, we obtained RNA expression values per cell types from Human Protein Atlas Dataset (proteinatlas.org). Single cell-type clusters were normalized separately from other transcriptomics datasets using trimmed mean of M values (TMM). To generate expression values per cell type, clusters were aggregated per cell type by first calculating the mean nTPM in all cells with the same cluster annotation within a dataset.

### Gene Set Enrichment Analysis

To further explore the potential function of the selected hub genes in AR, we used gene set enrichment analysis (GSEA_4.1.0) for single hub gene. In the dataset GSE138043, samples were divided into two groups according to the median expression level of hub genes. The h.all.v6.2.sytmbols.gmt in Molecular Signatures Database (MSigDB) was selected as the reference gene set, and adjusted p-value <0.05 was considered significantly different. The results were showed graphically by the R package “ggplot2.”

### Cell Culture

The Raw264.7 cells were cultured in Dulbecco’s modified Eagle’s medium (DMEM) High Glucose (11965084, Gibco) containing 10% fetal bovine serum (FBS) (12103C, Sigma) and 1% penicillin–streptomycin (15070063,Gibco), incubated at 37°C in a humidified atmosphere containing 5% CO_2_, and routinely passaged every 1 or 2 days. To induce RAW264.7 cell line to M1/M2 phenotypes, 10^5^ Raw264.7 cell were seeded in six-well plates 24 h before exposed to IFN-γ (20 ng/ml) + lipopolysaccharide (LPS) (10 ng/ml) to M1 phenotype, and IL-4 (10 ng/ml) to M2 phenotype for 24 h.

### Bone-Marrow-Derived Macrophages Isolation and Culture

Isolation and culture of bone-marrow-derived macrophages (BMDMs) were described by Pineda-Torra et al. (26). In brief, bone marrow was flushed out from the femurs and tibias, cultured and differentiated for 7 days in 1640 supplemented with 10% heat-inactivated FBS and 1% penicillin–streptomycin with macrophage-stimulating factor (M-CSF) (50 ng/ml) at 37°C in a humidified incubator with 5% CO_2_.

### Flow Cytometry to Measure Macrophage Polarization

Flow cytometry was used to measure the phenotypical changes in Raw264.7 macrophages. Single-cell suspension is prepared before staining with fluorochrome-labeled anti-CDF4/80 (clone BM8, BioLegend, San Diego, CA, USA), anti-CD80 (clone 16-10A1, eBioscience, San Diego, CA, USA). Data were analyzed using FlowJo v.10 (Treestar, Ashland, OR).

### Blood Sample and the Percutaneous Allograft Biopsy Collection

Research involving human participants was reviewed and approved by the Research Ethics Committee of the First Affiliated Hospital of Zhejiang University School of Medicine. Patients/participants (or their close relatives) provided written informed consent to participate in this study. Peripheral blood mononuclear cell (PBMC) was isolated within 3 h after collection. Percutaneous allograft biopsy was collected and fixed with 4% formalin for paraffin embedding. Biopsies were scored by the revised Banff 2019 classification of renal allograft pathology; rejection cases here were TCMR including borderline cases. **Supplementary Table S6** showed the characteristics of the samples used in this study.

### RNA Extraction and Real-Time Quantitative PCR

Total RNA was extracted from cultured cells and peripheral blood mononuclear cells (PBMCs) by Trizol reagent (Invitrogen, CA, USA). cDNA was prepared using the PrimeScriptTM RT Reagent Kit with gDNA Eraser (No. RR047A, Takara, Shiga, Japan) following the manufacturer’s protocol. Real-time PCR was run using SYBR Green and CFX96™ Real-Time PCR Detection Systems (Bio-Rad, CA, USA). The mRNA levels of selected genes were calculated after normalization to β-actin by using the 2^−ΔΔCt^ method according to the manufacturer’s protocol. All primer sequences used are shown in **Supplementary Table S4**.

### Immunohistochemistry

Immunohistochemistry (IHC) was performed following standard protocol. Briefly, after being dewaxed and rehydrated, the 2-μm paraffin-embedded sections were incubated with Anti-BLAME Polyclonal Antibody (bs-2473R, Bioss, Boston, MA, USA). After washing, the sections were incubated with the horseradish-peroxidase-labeled anti-mouse/rabbit IgG polymer (GK500710, GeneTech, South San Francisco, CA, USA) and diaminobenzidine. The sections were then counterstained with hematoxylin, dehydrated, and cleared. Six random fields of each section were photographed, and the staining was semi-quantified using the National Institutes of Health Image J by an investigator blinded to the experimental protocol.

### Immunofluorescence Assessment of Cultured Raw264.7

Coverslips containing RAW264.7 or BMDMs were fixed with 4% paraformaldehyde for 15 min and blocked in phosphate-buffered saline (PBS) containing 0.1% Triton X-100 and 3% bovine serum albumin(BSA) for 30 min at room temperature prior to incubation with Anti-BLAME Polyclonal Antibody (bs-2473R, Bioss), TLR4 antibody (sc-293072, Santa Cruz) overnight in a humidified chamber at 4°C. Slips were incubated with secondary antibody Alexa Fluor 594-conjugated anti-rabbit IgG (1:500) and Alexa Fluor 488-conjugated anti-mouse IgG (1:500) 1 h at 37°C. Sections were then examined by immunofluorescence microscopy (Leica DMLB, Wetzlar, Germany).

### Western Blotting

Total RAW264.7 cells were lysed in radioimmunoprecipitation assay (RIPA) cell lysis buffer following separated by sodium dodecyl sulfate–polyacrylamide gel electrophoresis (SDS-PAGE) and transferred to 0.22-μm polyvinylidene fluoride membranes (Millipore, Burlington, MA, USA). Blocked with 5% milk and incubated with the inducible nitric oxide synthase (iNOS) antibody (ab178945, Abcam, Cambridge, UK) at 4°C overnight. Protein was visualized using secondary anti-rabbit IgG or anti-mouse IgG (Sigma) with conjugated horseradish peroxidase and chemiluminescent substrate (Millipore, Billerica, MA, USA).

### Statistical Analysis

The statistical significance differences between the two groups were analyzed using non-parametric test or t-test based on data distribution characteristics. All analyses were conducted using software R4.1.0. A *p*-value < 0.05 was considered statistically significant.

## Results

### Differentially Expressed Genes Between AR and Non-AR Controls

**Figure 1:**
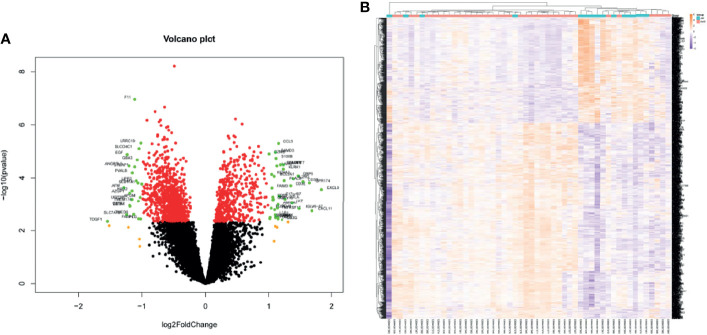
Screening for differentially expressed genes in the percutaneous allograft biopsy of 15 AR patients than NAR from GSE138043. **(A)** Differential genes in volcano plot. Red plots of adjusted *p*<0.01, orange plots of log2FC>1, green plots of both. **(B)** The clustering of differential genes in heatmap. The color in the heatmap represents the log2 expression values. Text on the right of heatmap indicates the enriched gene ontology terms for each cluster of genes. AR, rejection at 12 months post-renal transplant; NAR, non-rejection at 12 months post-renal transplant.

Kidney transcriptome data from GSE138043 containing 15 AR patients (rejection at 12 months post-renal transplant) and 37 NAR (non-rejection at 12 months post-renal transplant) was used for further analysis. We identified 1,561 DEGs (differential genes, adjusted *p*-values < 0.05) between the AR and NAR groups. Among 1,561 DEGs, 541 genes were upregulated, while 1,020 genes were downregulated. All genes are displayed in volcano plot in **Figure 1A**, and DEGs are listed in **Supplementary Table S1**. Additionally, the red plots represent adjusted *p* < 0.01, orange plots represent log2FC > 1, and green plots that were annotated represent both. Unsupervised clustering hierarchy was used in heatmap (**Figure 1B**).

### Weighted Co-expression Network Construction

**Figure 2:**
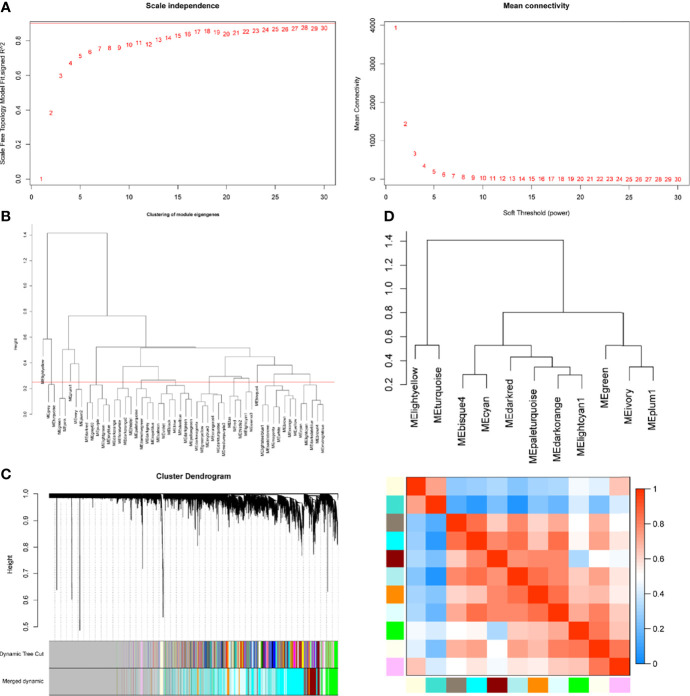
Determination of soft-threshold power in the WGCNA. **(A)** Left: Analysis of the scale-free topology model fit for various soft-threshold powers (β). Right: Analysis of the mean connectivity for various soft-threshold powers. **(B)** Clustering of module eigengenes. The red line represents MEDissThres=0.25. **(C)** Dendrogram of all differentially expressed genes clustered based on the measurement of dissimilarity (1-TOM). The branches correspond to modules of highly interconnected groups of genes. **(D)** The cluster dendrogram and adjacency heatmap of eigengenes.

WGCNA package was applied to compile the network. Keeping to the scale-free topology criterion, β = 17 was considered in this study for which the fit index curve flattens out upon reaching a high value (>0.9) and the mean connectivity ≤100 (**Figure 2A**). Hierarchical clustering was used to generate a hierarchical clustering dendrogram of genes; meanwhile, Dynamic Tree Cut R library was used for detecting clusters. As shown in **Figures 2B–D**, 12 distinct gene modules (M1–12) were defined, as MEDissThres was set to 0.25 to merge similar modules. Genes failing to fit within a distinct group were assigned to the gray module. The interaction relationships of 1,000 randomly selected genes were presented in the network heatmap (**Supplementary Figure S1A**).The genes in the same module were highly correlated, while they were weakly correlated to those in other modules. Thus, the reliability of the modules was verified.

### Identification of meta-modules and Hub Genes associated with AR

**Figure 3:**
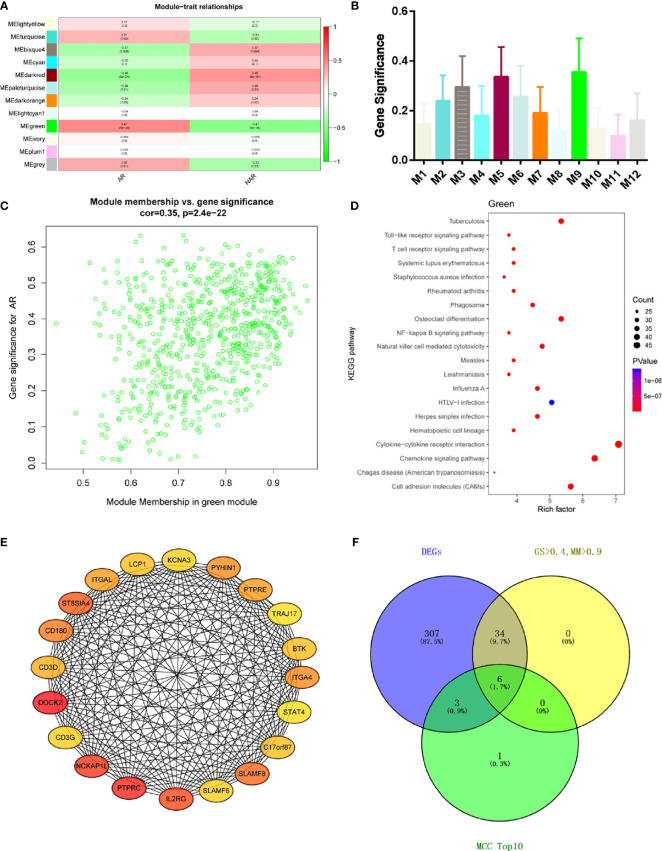
WGCNA revealed gene co-expression networks and the key genes in the percutaneous allograft biopsy of 15 AR patients. **(A)** Heatmap of the correlation between the module eigengenes and clinical traits of AR. We selected the green block for subsequent analysis. **(B)** Module significance values of those co-expression modules associated with SS (module significance value indicated the summary of gene significance of all genes in each module, and different colors of column indicated different modules). **(C)** The gene significance for AR in the green module (one dot represents one gene). **(D)** Top 20 pathways from Kyoto Encyclopedia of Genes and Genomes enrichment analysis. The x-axis represents KEGG enrichment scores, and the y-axis represents pathway terms. The colors of circle indicate *p*-values, and the size of circle indicates the numbers of differential RNAs. The redder and larger circle indicates that the enrichment of the pathway is higher and differential RNAs number is larger in the pathway. **(E)** Interaction of gene co-expression patterns in the green module. Each node corresponds to a gene. Colors from yellow to red correspond to the top 10 maximal clique centrality (MCC) values from low to high. **(F)** Identification of the hub gene in the intersection of MCC TOP10, DEGs, and GS > 0.4, and MM > 0.9.

Module-trait correlations analyses showed that multiple modules were related to AR (**Figure 3A**) The summary of significance of all genes in each module related to AR is shown in **Figure 3B**. Clearly, the green module was most significantly related to AR, followed by the turquoise module. **Figure 3C** shows the significance of these genes in the green module for AR (cor = 0.35, *p* = 2.4e−22). To investigate the potential biological function of genes in the green modules, we performed KEGG enrichment analysis. The top 20 pathways terms of green module are shown in **Figure 3D**. We found that genes in green modules played roles in cytokine–cytokine receptor interaction, chemokine signaling pathway, cell adhesion molecules (CAMs), tuberculosis, and other important regulatory processes. To identify hub genes in the target module, we calculated the MCC values *via* Cytohubba and constructed a network based on the top 10 genes (**Supplementary Table S2**). The module was visualized using Cytoscape 3.8 software (**Figure 3E**). The node colors coded from yellow to red (low to high) correspond to the top 10 MCC values from low to high. In intersection with GS > 0.4, MM > 0.9, and DEGs in the green module, six genes were regarded as the hub genes(**Figure 3F**), including dedicator of cytokinesis 2 (DOCK2), NCK-associated protein 1 like (NCKAP1L), interleukin 2 receptor subunit gamma (IL2RG), SLAM family member 8 (SLAMF8), CD180 molecule (CD180), and protein tyrosine phosphatase receptor type E (PTPRE). Relative mRNA expression of six hub genes in GSE138043 is shown in **Figure 1B**.

### GEO, Clinical, and qRT-PCR Validation

**Figure 4:**
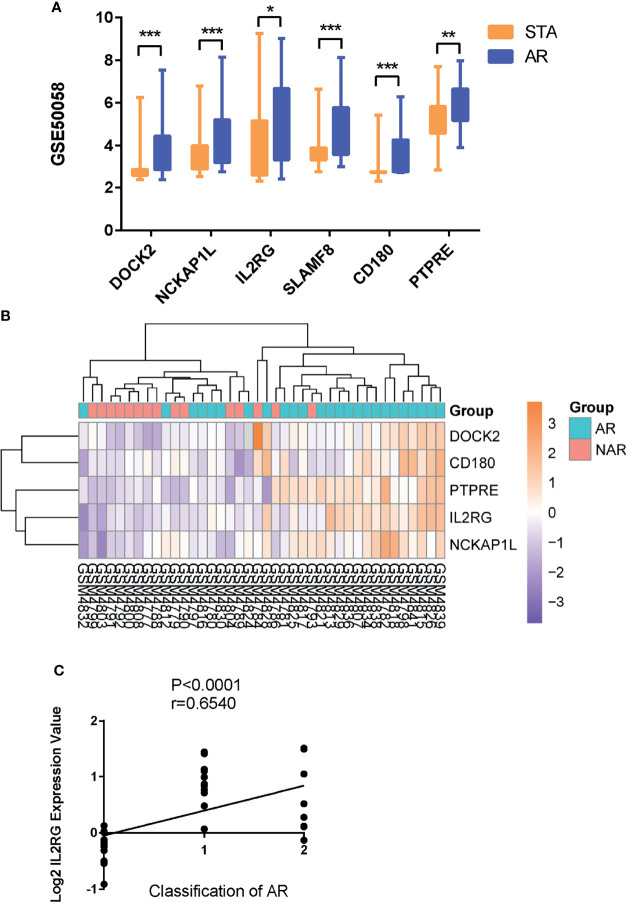
GEO and clinical validation. **(A)** Expression levels of DOCK2, NCKAP1L, IL2RG, SLAMF8, CD180, and PTPRE were significantly upregulated in the renal allograft biopsy of AR patient in dataset GSE50058. **(B)**The clustering of five hub genes in heatmap of dataset GSE343. **(C)** Correlation between the expression of IL2RG in AR with the Banff pathological grading of transplanted kidney. STA, stable patients; AR, patient with acute rejection. *p < 0.05, **p < 0.01, ***p < 0.0001.

As expected, the expression levels of hub genes including DOCK2, NCKAP1L, IL2RG, SLAMF8, CD180, and PTPRE were significantly upregulated in AR samples from the GSE50058 dataset (**Figure 4A**). For verifying hub genes, we obtained another dataset, GSE343, and analyzed the expression levels of the above five genes except for SLAMF8, which was not found in this dataset between AR patients and STA patients (**Figures 4B**). The expression of these genes was also upregulated in AR samples. We used the Nephroseq v5 online database to explore the correlation between the expression of IL2RG and clinical traits of AR. As shown, there was a positive correlation between the expression of IL2RG in AR with the Banff pathological grading of transplanted kidney (r=0.6540, *p<*0.0001) (**Figure 4C**).Correlation between the expression of DOCK2 in AR and the Banff pathological grading is shown in **Supplementary Figure S1C** (r=0.4863, *p<*0.0035).

**Figure 5:**
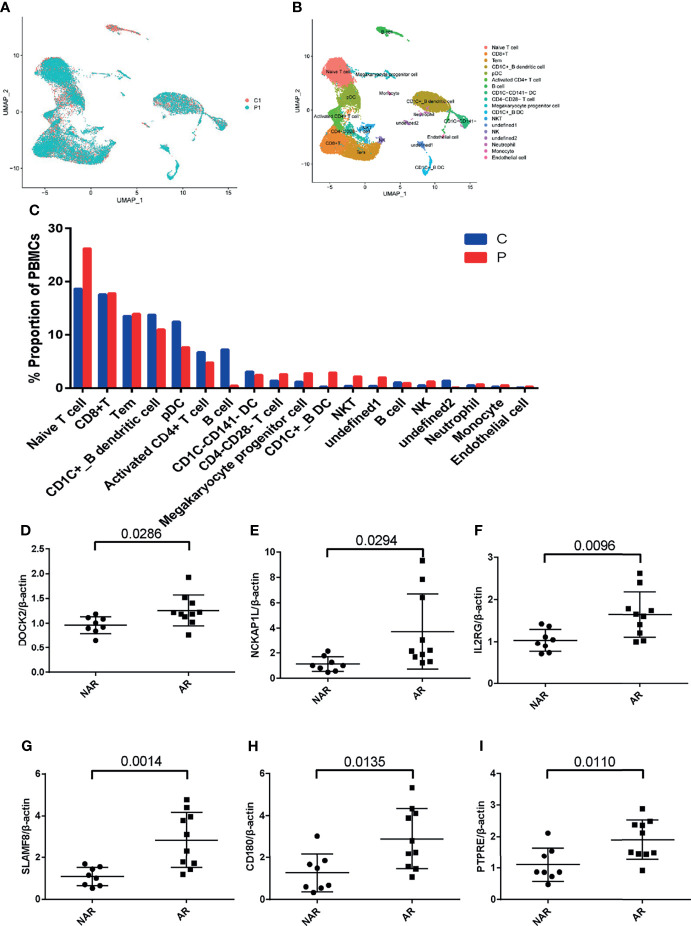
scRNA-seq in patient PBMCs of acute rejection post renal transplant and qRT-PCR validation. **(A)** Uniform manifold approximation and projection (UMAP) of 26,192 cells, split between P1 and C1. **(B)** UMAP plot of 19 cell clusters identified based on the expression of highly variable genes. **(C)** Single-cell RNA sequencing revealed a higher percentage of native T, CD1C+_B DC, NKT, NK, and monocytes in P1 compared with C1. Relative mRNA expression of DOCK2 **(D)**, NCKAP1L **(E)**, IL2RG **(F)**, SLAMF8 **(G)**, CD180 **(H)**, and PTPRE **(I)** were measured in peripheral blood mononuclear cells (PBMCs)of 8 NAR and 10 AR patients. Data shown are mean ± SD by an unpaired t-test; P1, patient of acute rejection post renal transplant; C1, control patient of stable kidney function post renal transplant; NAR, non-acute rejection; AR, acute rejection.

To study the transcriptional changes related to AR further, we performed single-cell RNA sequencing in P1 (patient PBMCs of acute rejection post renal transplant) and C1 (control patient of stable kidney function post renal transplant). The patient had a higher percentage of native T, CD1C+_B DC, NKT, NK, and monocytes in P1 compared with C1 (**Figures 5A–C**). Expression of DOCK2, NCKAP1L, IL2RG, SLAMF8, CD180, and PTPRE (colored single cells) on UMAP plot projecting PBMCs from P1 and C1 are shown in **Supplementary Figure S2**. In addition, to further validate these six hub genes, we collected PBMCs from eight non-AR and 10 AR patients to perform qRT-PCR. The results showed that compared with NAR group, the mRNA levels of DOCK2, NCKAP1L, IL2RG, SLAMF8, CD180, and PTPRE were all significantly elevated in AR patients (**Figures 5D–I**).

### Cell-Type Enrichment Analysis

**Figure 6:**
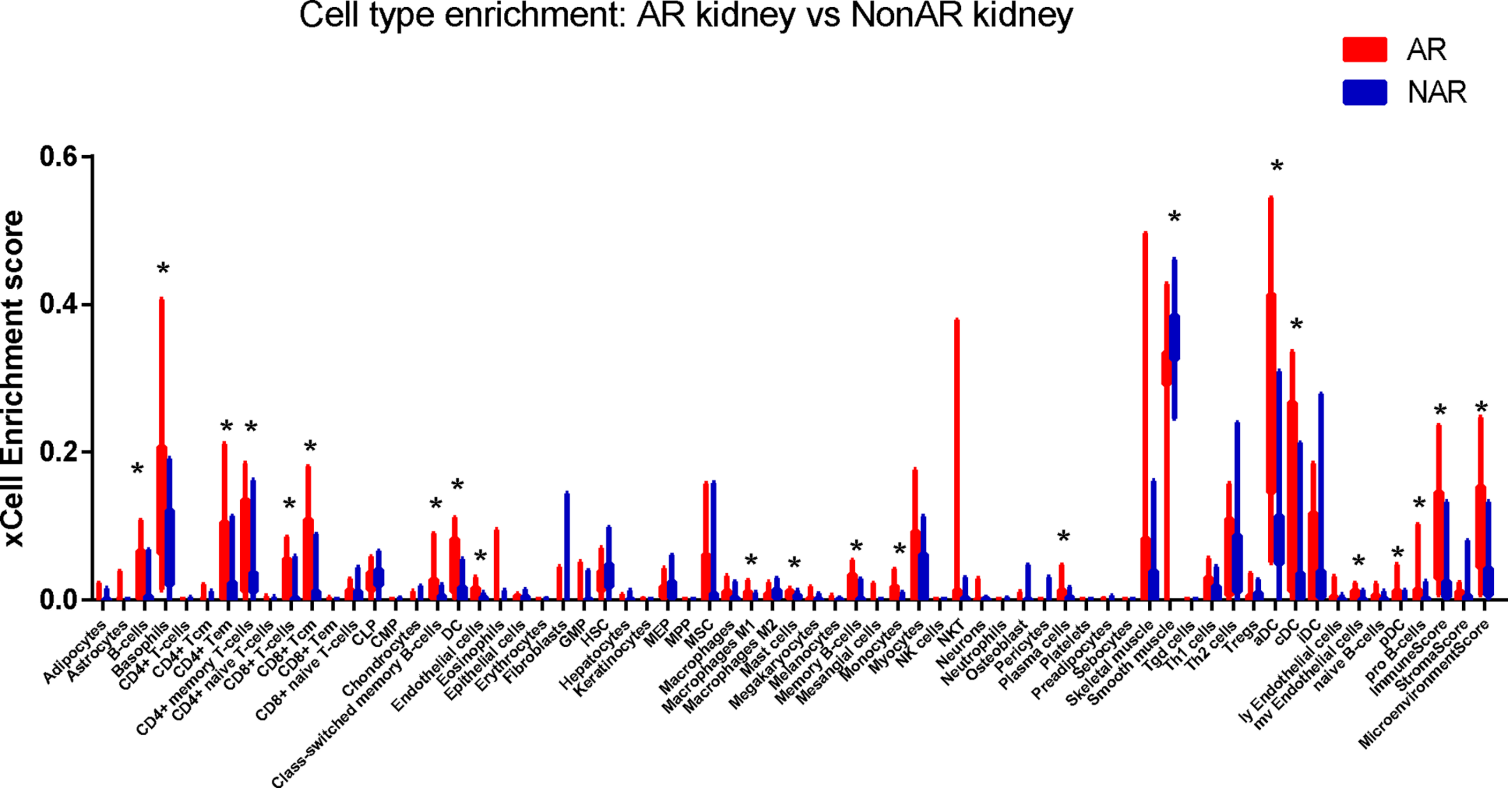
Immune cells enrichment. Distribution of cell-type enrichment scores for AR and NAR. xCell, a bioinformatics tool, was used to provide an enrichment score for different cell types that allow comparison of cell types across group. The x-axis represents cell types. The y-axis represents the xCell enrichment score. *p < 0.01.

Cell-type enrichment analysis was performed by xCell from gene expression data for 64 immune and stroma cell types. Our data revealed that the AR group had higher immune scores and microenvironment scores than the NAR group. Among the 64 cell types, scores of 20 types of cells were significantly differently expressed (*p*<0.01), including immune cells, such as B cells, basophils, CD4+ Tem, CD4+ memory T cells, CD8+ T cells, CD8+ Tcm, class-switched memory B cells, DC, macrophages, M1, mast cells, memory B cells, monocytes, plasma cells, aDC, cDC, naive B cells, pDC, and stroma cells such as endothelial cells, smooth muscle, and MV endothelial cells (**Figure 6**). We used the Protein Atlas online database to explore the six hub genes RNA expression in 76 single-cell types (**Supplementary Figure S3**). Except for SLAMF8, which is more singly expressed in Langerhans cell and macrophages, the other five genes are more widely expressed in immune cells such as B cells, DC, T cells, and monocyte consistent with the different cell types in AR groups, meaning that six hub genes exercise certain biological properties in these cell types during AR.

### Gene Set Enrichment Analysis

**Figure 7:**
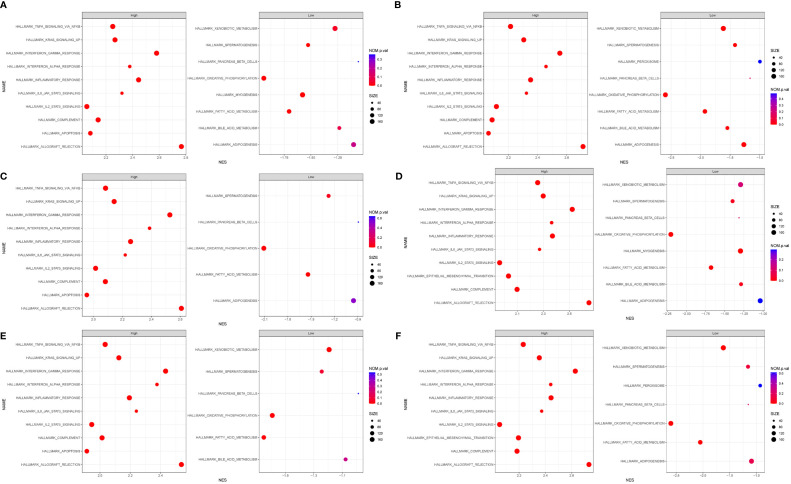
Gene set enrichment analysis (GSEA). The full list of gene sets enriched in samples with DOCK2 **(A)**, NCKAP1L **(B)**, IL2RG **(C)**, SLAMF8 **(D)**, CD180 **(E)**, and PTPRE **(F)** highly expressed.

We performed GSEA, and h.all.v7.4.sytmbols.gmt in MSigDB was used as the reference gene set. The full list of gene sets enriched in samples with DOCK2 (**Figure 7A**), NCKAP1L (**Figure 7B**), IL2RG (**Figure 7C**), SLAMF8 (**Figure 7D**), CD180 (**Figure 7E**), or PTPRE (**Figure 7F**) highly expressed is shown. Four gene sets were enriched in samples with highly expressed DOCK2, NCKAP1L, IL2RG, SLAMF8, CD180, and PTPRE, including “allograft rejection”, “interferon γ response”, “interferon α response”, and “inflammatory response”. Moreover, gene sets “IL6–JAK–STAT3 signaling”, “KRAS signaling”, and “TNFα signaling *via* NFkB” were enriched in the samples with either DOCK2, NCKAP1L, IL2RG, SLAMF8, CD180, and PTPRE highly expressed.

### SLAMF8 Participate in AR Progression *via* TLR4 *In Vivo*

**Figure 8:**
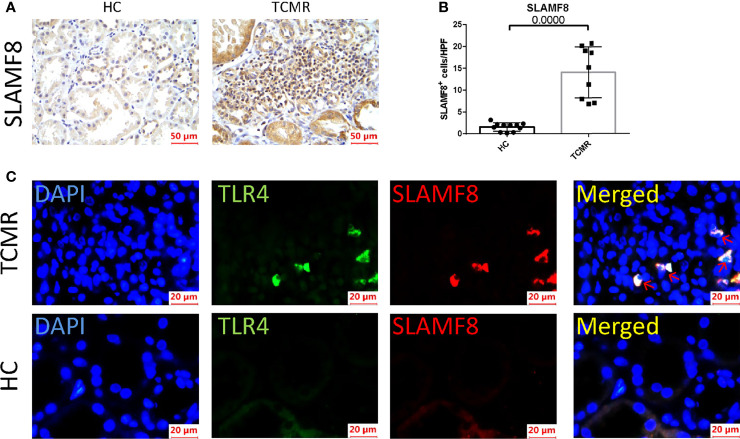
SLAMF8 participate in AR progression *via* TLR4 *in vivo*. **(A, B)** Representative images and quantification of SLAMF8^+^ cells number in the human allograft diagnosed as TCMR (n=9) clinically and HC (n=11). Scale bar: 50 μm; six random fields were taken from each kidney. **(C)** Representative of immunofluorescence staining of TLR4 (green) and SLAMF8 (red) in TCMR (n=11) and HC (n=8). Red arrow indicates cells co-expressing TLR4 and SLAMF8. Scale bar = 20μm; TCMR, T-cell-mediated rejection; HC, healthy control.

SLAMF8, one of the six hub genes, was also the top rejection-associated transcripts in TCMR versus everything else including ABMR in previous study (27). In addition, SLAMF8 is more specifically expressed in macrophages, which is exactly the cell type that differentially infiltrated in the AR group in our above research results (**Figure 6**; **Supplementary Figure S3D**). Immunohistochemical staining of kidney tissue revealed a higher level of SLAMF8 expression in the renal interstitium from TCMR (T-cell-mediated acute rejection after renal transplantation) kidney tissue than from HC (healthy donor control) (**Figures 8A, B****)**. A previous study confirmed that SLAMF8 maintained TLR4 expression on macrophages and promoted LPS-induced mitogen-activated protein kinase (MAPK) activation (28). We subsequently determined whether the SLAMF8 participates in macrophage activation *via* TLR4. Fluorescence detection of SLAMF8 and TLR4 revealed that approximately complete SLAMF8+ cells express TLR4 (**Figure 8C**). Thus, we speculated that SLAM8 participates in AR progression, likely through upregulating TLR4 expression.

### SLAMF8 and TLR4 Are Co-expressed in M1-Type Macrophages

**Figure 9:**
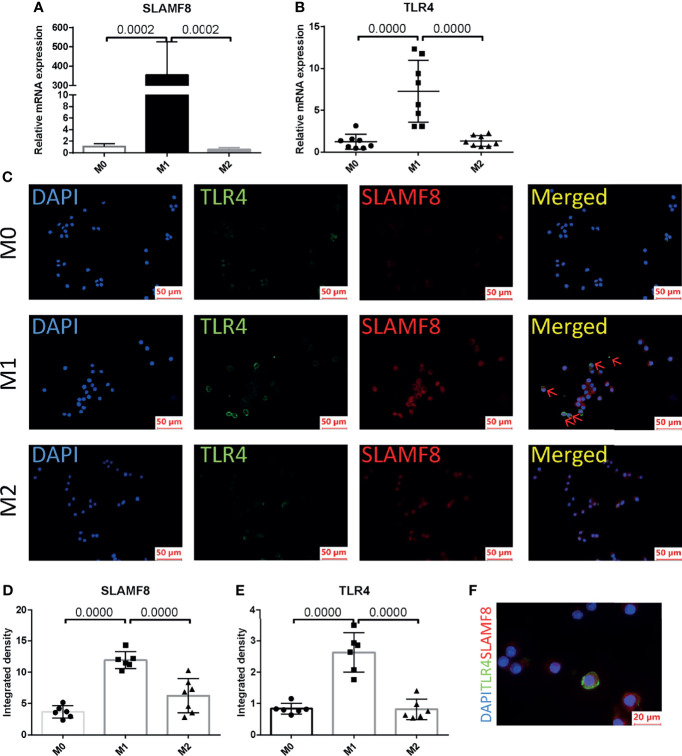
SLAMF8 and TLR4 are co-expressed in M1-type macrophages. Relative mRNA expression of SLAMF8 **(A)** and TLR4 **(B)** were measured in M0, M1, and M2. **(C–E)** Representative and quantification of immunofluorescence staining of TLR4 (green) and SLAMF8 (red) in M0, M1, and M2. Red arrow indicates cells co-expressing TLR4 and SLAMF8. Scale bar = 50μm; six random fields were taken from each coverslip (mean ± SD, n = 6). **(F)** Representative of immunofluorescence staining of TLR4 (green) and SLAMF8 (red) in M1. Scale bar = 20μm. M0, murine RAW 264.7; M1, RAW 264.7 treated with 10 ng/ml LPS plus 20 ng/ml IFNγ for 24 h; M2, RAW 264.7 treated with 10 ng/ml IL-4 for 24 h.

In order to explore in which phenotype of macrophages SLAMF8 is specifically expressed, murine RAW 264.7 macrophages and primary BMDM cells were treated with 10 ng/ml LPS plus 20 ng/ml IFNγ or 10 ng/ml IL-4 for 24 h. CD80 and CD206 are known to be a specific surface marker of the M1 phenotype and the M2 phenotype, respectively. Flow cytometry and RT-qPCR were used to measure the polarization of RAW 264.7 macrophages (**Supplementary Figures S4A–D**). Western blotting using the M1 phenotype marker iNOS was confirmed to be increased in the LPS plus IFNγ treated group in RAW264.7 (**Supplementary Figure S4E**). The mRNA expression of SLAMF8 and TLR4 was increased in LPS and IFNγ treated RAW 264.7 macrophages and BMDMs (**Figures 9A, B**; **Supplementary Figures S5A, B**) consistent with the phenomenon in immunofluorescence staining (**Figures 9C–E**; **Supplementary Figures S5C–E**). Two-color immunofluorescence analysis revealed that the M1 phenotype contained large numbers of TLR4-expressing SLAMF8+ cells in contrast to M0- and M2-type macrophages (**Figures 9C–F**; **Supplementary Figures 5C–E**).

## Discussion

Even if the incidence of clinical acute rejection and subclinical rejection in the first year after kidney transplantation is controlled in 10%–15%, respectively, preventing acute rejection remains the key to achieving long-term graft survival (6). Therefore, timely detection of rejection is important for the surveillance after transplantation. To our knowledge, there are few studies analyzing kidney transplant rejection using WGCNA (20, 21, 29). This study shows that co-expression network analysis was employed to mine the hub gene based on expression in the percutaneous allograft biopsy of 15 AR and 37 NAR. Through WGCNA, we divided all genes into 12 separate modules and found that the green module was the most related to AR. Six hub genes were then screened out satisfying three criteria: (1) DEGs; (2) GS > 0.4, MM > 0.9; and (3) the 10 highest MCC value, followed by validation in both two datasets and clinical traits of AR. Of the six hub genes derived, some have well-described functions in the immune response (e.g., DOCK2, IL2RG, PTPRE, and CD180), while others have not been as well characterized (e.g., NCKAP1L and SLAMF8) and represent opportunities for future study.

DOCK2, a member of the CDM protein family, plays a critical role in lymphocyte homing and immunological synapse formation by remodeling the actin cytoskeleton in response to chemokine signaling (30). DOCK2 mediates GTP–GDP exchange reaction for Rac through its DOCK homology region (DHR)-2 (also known as Docker) domain (31). Mutations in this gene result in immunodeficiency, a combined form of immunodeficiency that affects T-cell number and function, also with variable defects in B-cell and NK-cell function (32, 33). Deletion of DOCK2 suppresses cardiac allograft rejection (34). IL2RG, interleukin 2 receptor subunit gamma chain, or CD132, is the co-receptor subunit of a variety of important immune factors, including IL-2, IL-4, IL-7, IL-9, IL-15, and IL-21. Therefore, it is also called the receptor common gamma chain (γc) (35). In mammals, the IL2Rg gene is located on the X chromosome. Its mutation called X-linked severe combined immunodeficiency (X-SCID), presenting with absent or profoundly diminished peripheral T and NK cells and functionally defective B cells (36, 37). In severe combined immunodeficiency (SCID) gene homozygous mutation or recombination activation gene 1 (Rag1) or Rag2 homozygous mutation mice, accompanied by mutation of the interleukin 2 receptor gamma chain (IL2Rg) locus, comparing with previous immunodeficiency mouse models, the implantation and function of human hematopoietic stem cells (HSC) and peripheral blood mononuclear cells (PBMC) are greatly increased (38). In this study, we found that DOCK2 and IL2RG mRNA levels correlated to inflammatory parameters according to the Banff classification.

The nine receptors of the SLAM family are differentially expressed on the surface of hematopoietic cells such as thymocytes, memory CD4+ and CD8+T cells, dendritic cells, monocytes, macrophages, and platelets. They regulate not only the proliferation, cytotoxicity, and cytokine production of T lymphocytes but also the lytic activity, cytokine production, and MHC-independent inhibition of natural killer (NK) cells; B cell activation and proliferation; regulation of neutrophil; and macrophage killing and platelet aggregation (39). In contrast to the classical SLAMF receptors, SLAMF8 have no signaling motifs including the ITSM in their short cytoplasmic tail. Limited studies have indicated that combined deficiency of SLAMF8 and SLAMF9 prevents endotoxin-induced liver inflammation by downregulating TLR4 expression on macrophages (28), and SLAMF8 can negatively regulate ROS production by macrophages (40). In addition, a previous study has shown that SLAMF8 is the top differentiating transcripts rejection-associated transcripts in TCMR versus everything else including ABMR, and SLAMF8 is the most specific transcript in macrophages cell lines for TCMR (27, 41). Bone marrow cells follow a differentiation trajectory from monocytes to pro-inflammatory macrophages and become predominantly infiltrating cells in the intimal arteritis of biopsies graded as Banff II or III acute allogeneic renal rejection (42, 43). Due to SLAMF8 mainly expressed on macrophages and the important role of macrophages in transplant rejection, we further investigated the expression of SLAMF8 in from TCMR *in vivo* and macrophages of different phenotypes *in vitro*. We observed that SLAMF8 expressed highly in TCMR than HC and in M1 phenotype.

TLR4 is a member of the toll-like receptor family and plays an important role in regulating innate immunity in response to exogenous and endogenous molecular patterns (44). Activation of the innate immunity through toll-like receptors (TLRs) has been postulated to play an important role in the pathophysiology of renal allograft dysfunction (45). Renal transplant recipients with TLR4 polymorphism present a lower risk of acute allograft rejection and lower rates of delayed graft function as compared to those with normal TLR4 function (46), also consistent with the observation that acute kidney allograft rejection was modestly attenuated in TLR4^−/−^ mice (47). Previous reports have demonstrated that activation of TLR4 triggers a phenotypic switch of macrophages from a quiescent population to an inflammatory population and inhibition of TLR4 suppressed macrophages polarization (48–50). Because SLAMF8 has no signaling activity and the TLR4 has essential role in macrophages and immune rejection, we wondered whether SLAMF8 participates in macrophage activation *via* TLR4. Here, we identified that approximatively complete SLAMF8+ cells express TLR4 in TCMR. M1 macrophage contained large numbers of TLR4-expressing SLAMF8+ cells in contrast to M0- and M2-type macrophages.

In summary, our study finds involvement of the key gene co-expression module, hub genes, and some functional biological pathways related to “interferon γ response”, “interferon α response”, and “inflammatory response” in the pathogenesis of AR. SLAMF8 was highly expressed in pro-inflammatory macrophage-mediated acute renal transplantation rejection accompanied by TLR4 high expression, and it presents a potentially novel therapeutic target for controlling kidney allograft rejection and improving kidney allograft survival. These findings provide new insights into the development of AR, although the exact molecular mechanism of hub genes and functional pathway in AR still need to be further explored.

## Data Availability Statement

The data presented in the study are deposited in the OMIX, China National Center for Bioinformation / Beijing Institute of Genomics, Chinese Academy of Sciences (https://ngdc.cncb.ac.cn/omix/release/OMIX873), accession number OMIX873.

## Ethics Statement

The studies involving human participants were reviewed and approved by the Research Ethics Committee of the First Affiliated Hospital, College of Medicine, Zhejiang University. The patients/participants provided their written informed consent to participate in this study.

## Author Contributions

HJ, JC, and LT designed the study. LT and LS analyzed bioinformatics. CW, SF, and YW collected PBMC and the percutaneous allograft biopsy from AR patients and healthy donor. WZ and YB carried out experiments. SR, NS, and HH analyzed data. All authors contributed to the article and approved the submitted version.

## Funding

This work was supported by NSFC81970651, U21A20350, Sino-German Center GZ1572.

## Conflict of Interest

The authors declare that the research was conducted in the absence of any commercial or financial relationships that could be construed as a potential conflict of interest.

## Publisher’s Note

All claims expressed in this article are solely those of the authors and do not necessarily represent those of their affiliated organizations, or those of the publisher, the editors and the reviewers. Any product that may be evaluated in this article, or claim that may be made by its manufacturer, is not guaranteed or endorsed by the publisher.

## Supplementary Material

Supplementary Figure 1 **(A)** Network heatmap plot in the co-expression modules (The progressively saturated red colors indicated higher overlap among the functional modules). **(B)** Relative mRNA expression of six hub genes in AR and NAR tissues. NAR: Non rejection at 12 months post renal transplant; AR: Rejection at 12 months post renal transplant. Box represents mean ± SD by an unpaired t-test. *P < 0.05, **P < 0.01, ***P < 0.0001, ns, no significance **(C)** Correlation between the expression of DOCK2 in AR and the AR classification.

Supplementary Figure 2 Visualization of expression of DOCK2, NCKAP1L, IL2RG, SLAMF8, CD180 and PTPRE (coloured single cells) on UMAP plot projecting PBMCs from P1 (*n* = 14,118 cells) and C1 (*n* = 12,074 cells). P1: patient of acute rejection post renal transplant; C1: control patient of stable kidney function post renal transplant.

Supplementary Figure 3 Six hub genes RNA expression in Top 15 single cell types. DOCK2 **(A)**, NCKAP1L **(B)**, IL2RG **(C)**, SLAMF8 **(D)**, CD180 **(E)** and PTPRE**(F)**. Data were obtained from Human Protein Atlas Dataset available from proteinatlas.org.

Supplementary Figure 4 Relative mRNA expression of CD80 **(A)** iNOS **(B)** and CD206 **(C)** were measured in M0, M1 and M2. **(D)** Representative histograms of CD80 expression among M0, M1 and M2 in flow cytometry. **(E)** Mean fluorescence intensity of CD80 in M0, M1 and M2. **(F)** Western blotting of iNOS in M0, M1 and M2. M0: murine RAW 264.7, M1: RAW 264.7 treated with 10ng/ml LPS plus 20ng/ml IFNγ for 24 h, M2: RAW 264.7 treated with 10ng/ml IL-4 for 24h.

Supplementary Figure 5 SLAMF8 and TLR4 are co-expressed in LPS plus IFNγ treated BMDMs. Relative mRNA expression of SLAMF8 **(A)** and TLR4 **(B)** were measured in BMDMs, BMDMs+LPS (10ng/ml)+IFNγ (20ng/ml) and BMDMs+IL-4(10ng/ml) for 24 h. **(C–E)** Representative and quantification of Immunofluorescence staining of TLR4(green) and SLAMF8(red) in BMDMs, BMDMs+LPS+IFNγ and BMDMs+IL-4. Scale bar = 50μm; Six random fields were taken from each coverslip(mean ± SD, n = 6).

Supplementary Table 1 DEGs in GSE138043.

Supplementary Table 2 The MCC values of the top 10 genes in the green module.

Supplementary Table 3 Cell-type enrichment score.

Supplementary Table 4 All primer sequences.
