# Supplementary figures and images for: SLAMF8 Participates in Acute Renal Transplant Rejection via TLR4 Pathway on Pro-Inflammatory Macrophages
Source: Front Immunol. 2022 Apr 1;13:846695. doi: 10.3389/fimmu.2022.846695 (PMC9012444; doi:10.3389/fimmu.2022.846695)

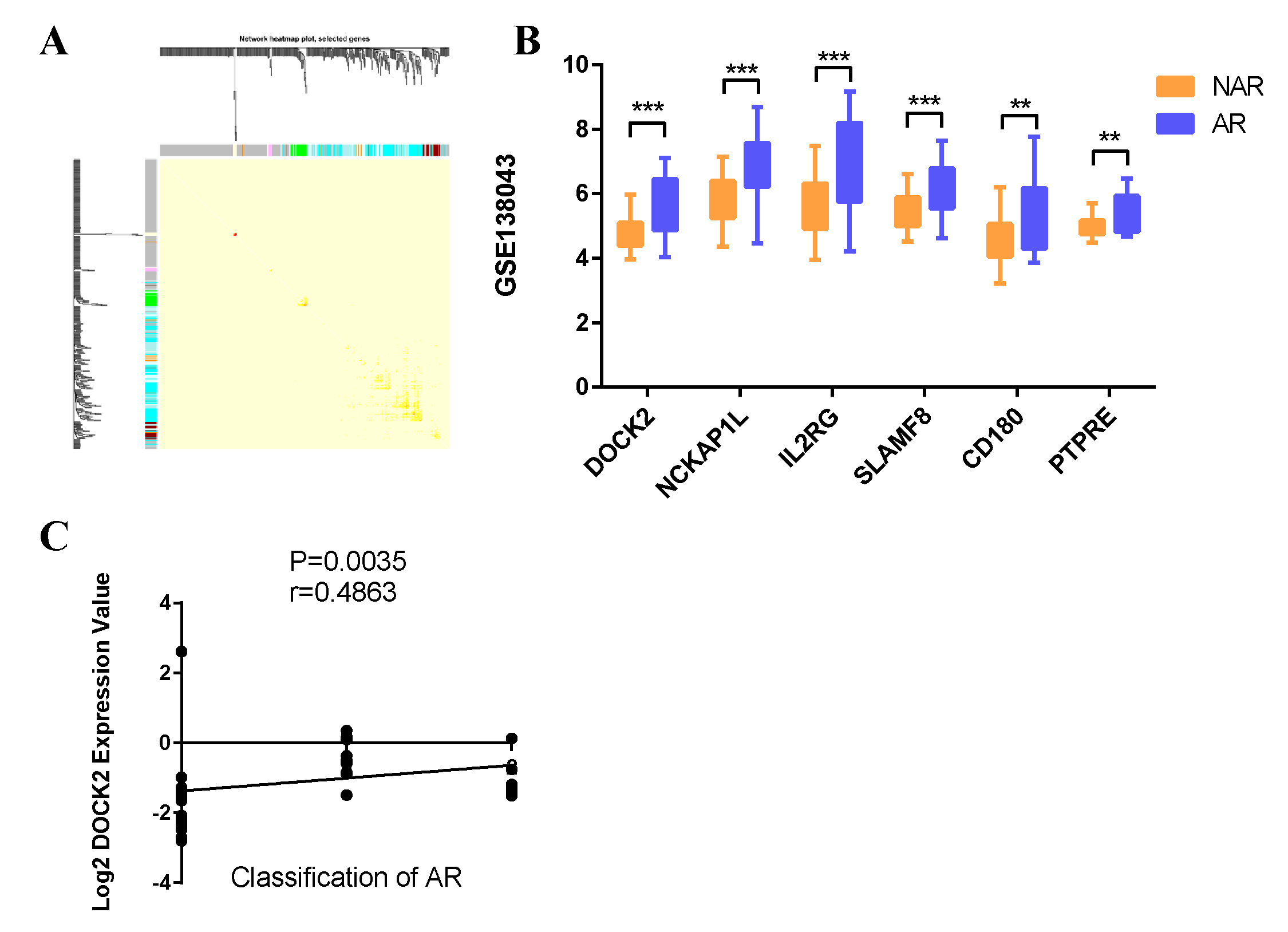

Supplement: Supplementary Figure 1 — (A) Network heatmap plot in the co-expression modules (The progressively saturated red colors indicated higher overlap among the functional modules). (B) Relative mRNA expression of six hub genes in AR and NAR tissues. NAR: Non rejection at 12 months post renal transplant; AR: Rejection at 12 months post renal transplant. Box represents mean ± SD by an unpaired t-test. *P < 0.05, **P < 0.01, ***P < 0.0001, ns, no significance (C) Correlation between the expression of DOCK2 in AR and the AR classification. [file Image_1.tif]

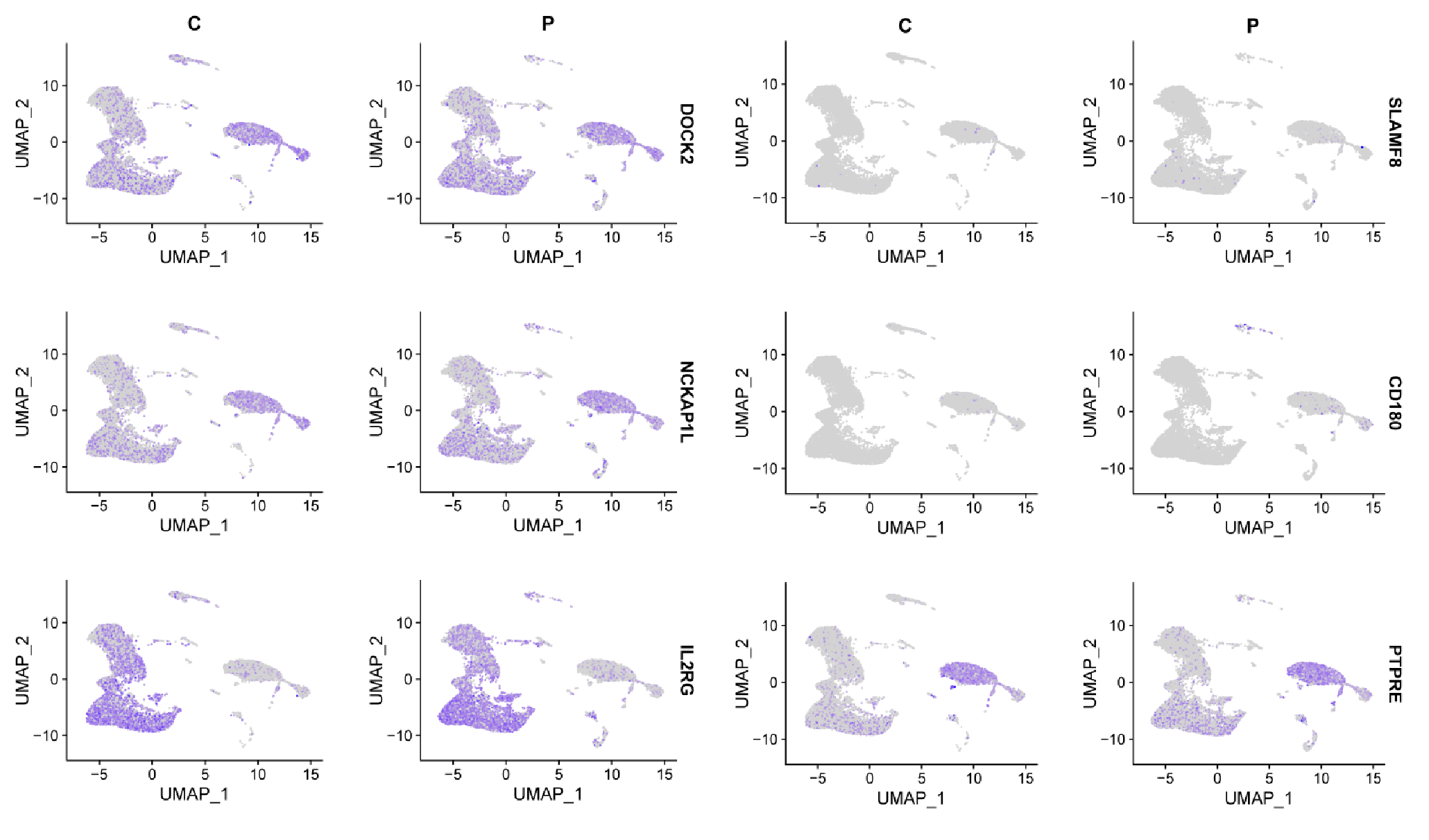

Supplement: Supplementary Figure 2 — Visualization of expression of DOCK2, NCKAP1L, IL2RG, SLAMF8, CD180 and PTPRE (coloured single cells) on UMAP plot projecting PBMCs from P1 (n = 14,118 cells) and C1 (n = 12,074 cells). P1: patient of acute rejection post renal transplant; C1: control patient of stable kidney function post renal transplant. [file Image_2.tif]

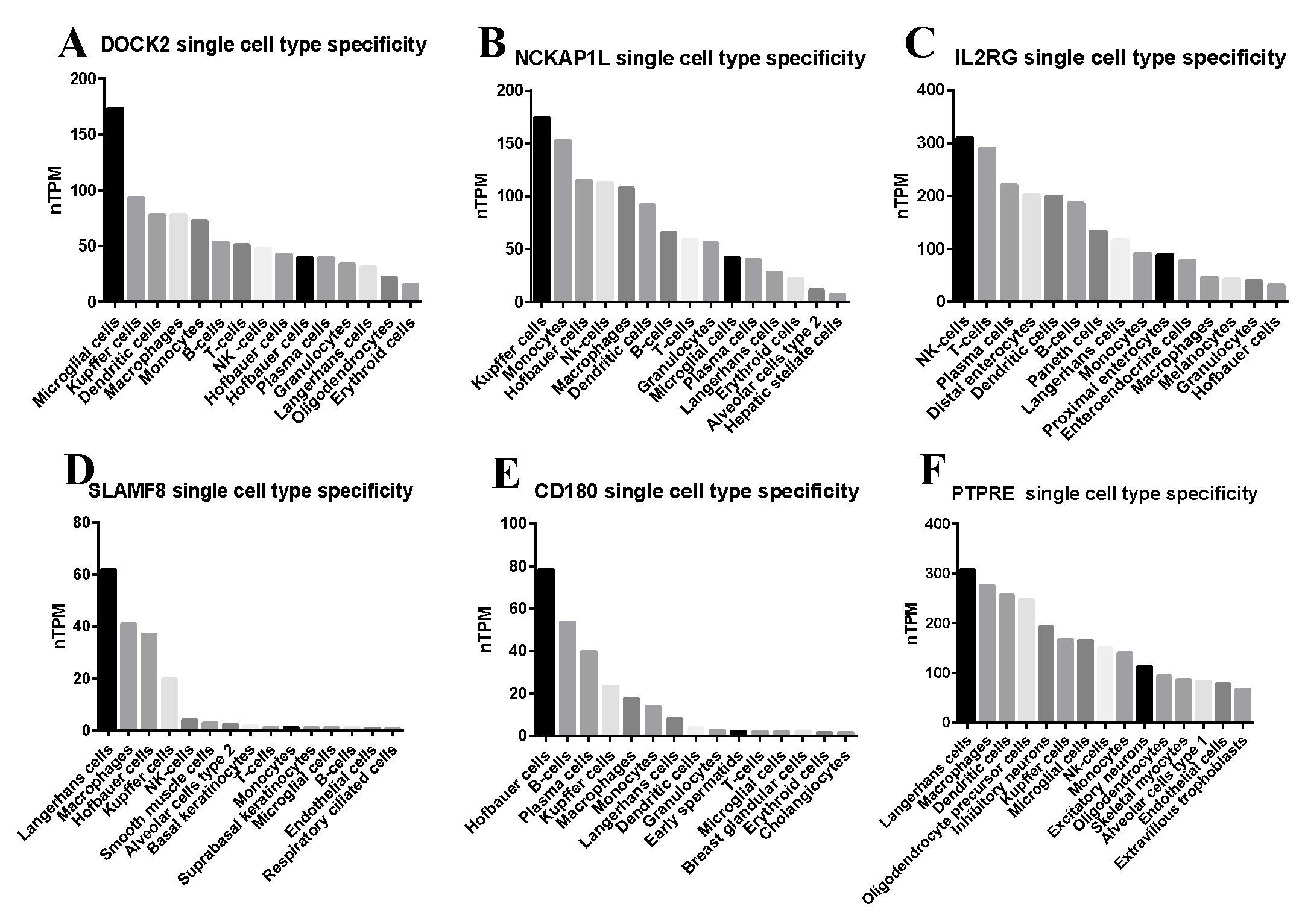

Supplement: Supplementary Figure 3 — Six hub genes RNA expression in Top 15 single cell types. DOCK2 (A), NCKAP1L (B), IL2RG (C), SLAMF8 (D), CD180 (E) and PTPRE(F). Data were obtained from Human Protein Atlas Dataset available from proteinatlas.org. [file Image_3.tif]

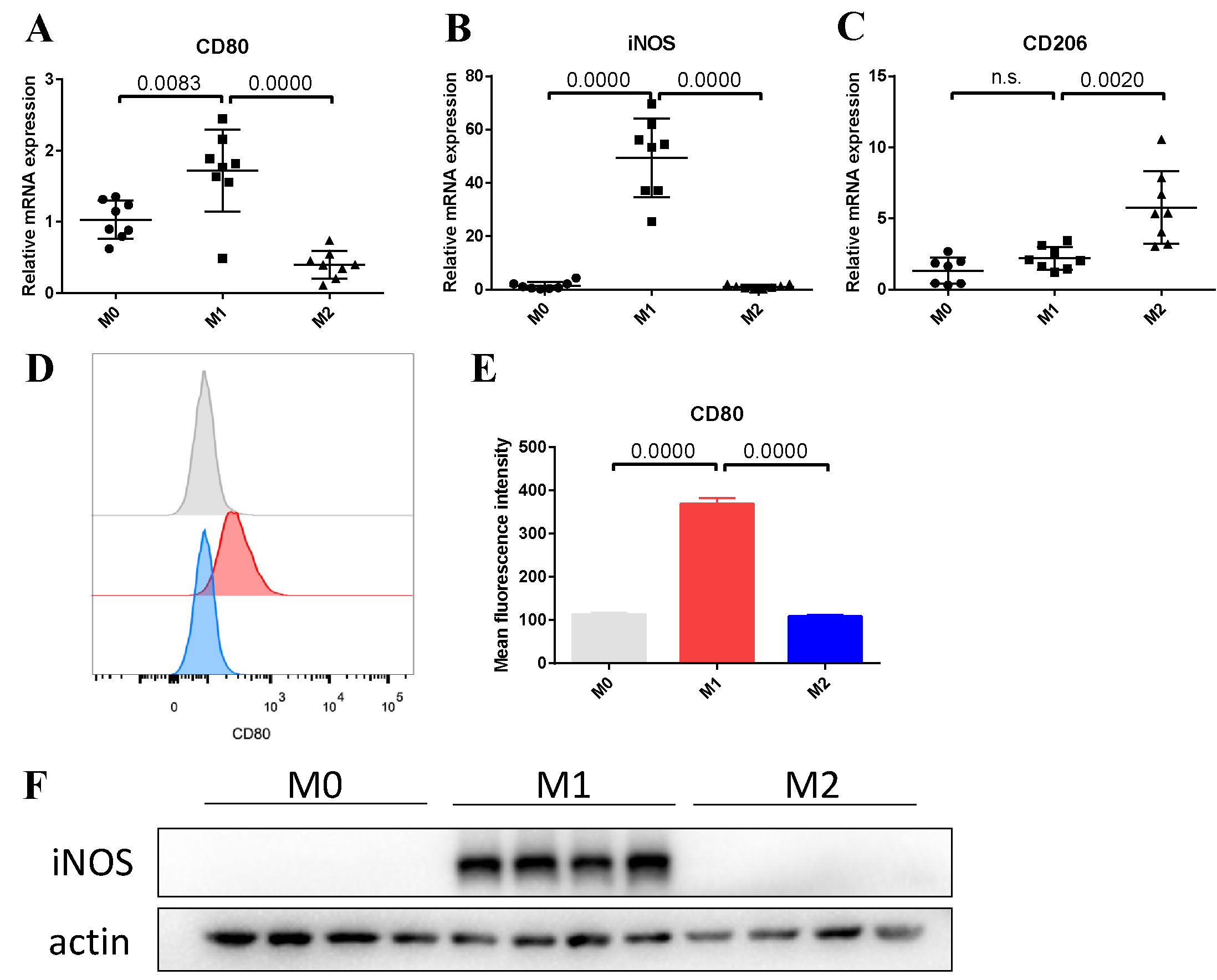

Supplement: Supplementary Figure 4 — Relative mRNA expression of CD80 (A) iNOS (B) and CD206 (C) were measured in M0, M1 and M2. (D) Representative histograms of CD80 expression among M0, M1 and M2 in flow cytometry. (E) Mean fluorescence intensity of CD80 in M0, M1 and M2. (F) Western blotting of iNOS in M0, M1 and M2. M0: murine RAW 264.7, M1: RAW 264.7 treated with 10ng/ml LPS plus 20ng/ml IFNγ for 24 h, M2: RAW 264.7 treated with 10ng/ml IL-4 for 24h. [file Image_4.tif]

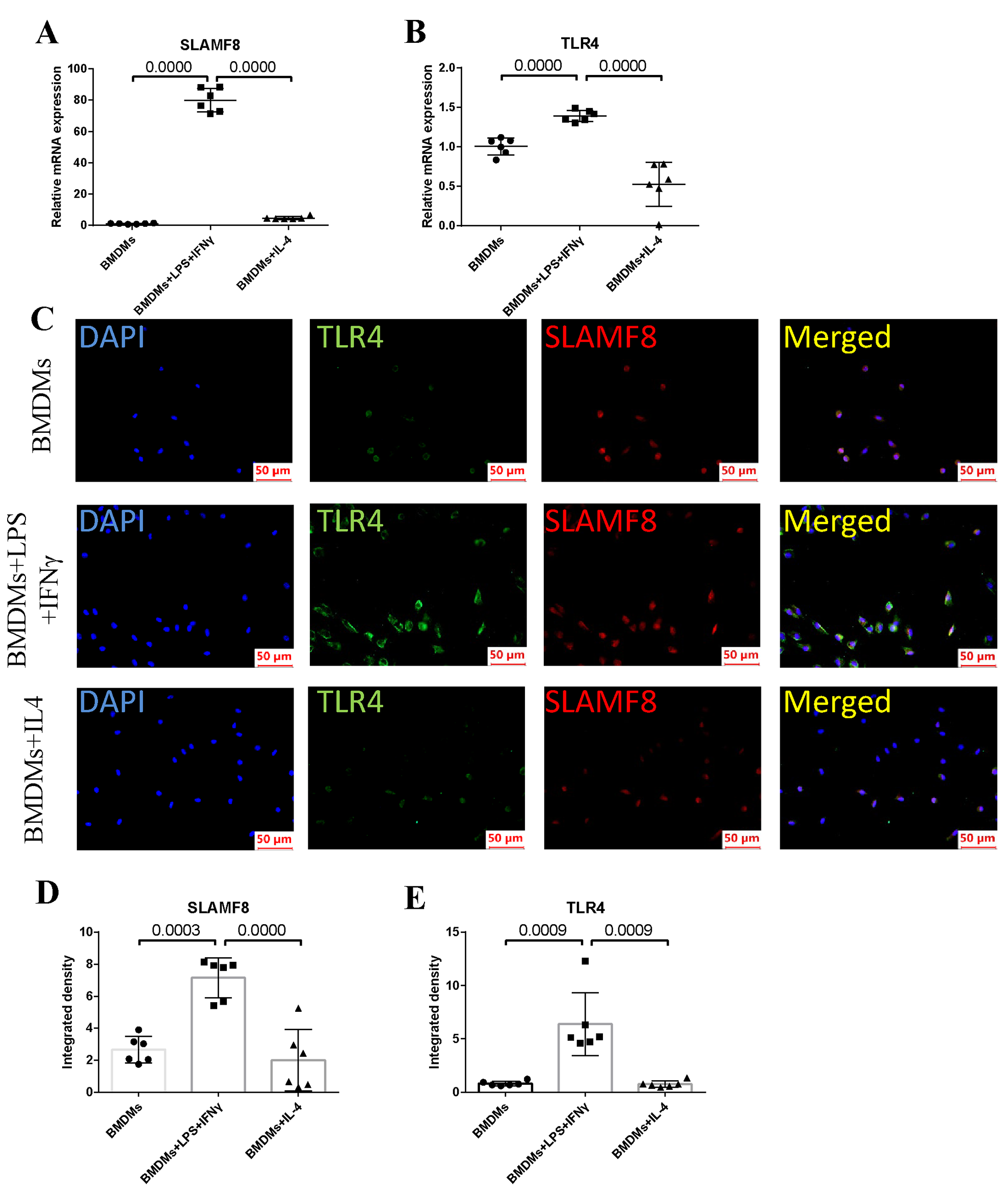

Supplement: Supplementary Figure 5 — SLAMF8 and TLR4 are co-expressed in LPS plus IFNγ treated BMDMs. Relative mRNA expression of SLAMF8 (A) and TLR4 (B) were measured in BMDMs, BMDMs+LPS (10ng/ml)+IFNγ (20ng/ml) and BMDMs+IL-4(10ng/ml) for 24 h. (C–E) Representative and quantification of Immunofluorescence staining of TLR4(green) and SLAMF8(red) in BMDMs, BMDMs+LPS+IFNγ and BMDMs+IL-4. Scale bar = 50μm; Six random fields were taken from each coverslip(mean ± SD, n = 6). [file Image_5.tif]
